# Supplementary material for: Mixed Neuroendocrine Non-Neuroendocrine Neoplasms: A Systematic Review of a Controversial and Underestimated Diagnosis
Source: J Clin Med. 2020 Jan 19;9(1):273. doi: 10.3390/jcm9010273 (PMC7019410; doi:10.3390/jcm9010273)
Supplement: Supplementary file 1 [file jcm-09-00273-s001.pdf]

Supplementary Materails:

Table S1. Survival outcomes of patients with a diagnosis of mixed neuroendocrine non-neuroendocrine neoplasms in retrospective studies where median survival estimates by Kaplan-Meier analysis were not reached.

Reference	Total N patients	Median follow-up time	Localised stage	Advanced stage
Nie L., 2016	14	17 m	• 4 recurred and died after 8, 8, 10 and 11m<br>• 8 free from recurrence and alive after 9, 12, 12, 12, 28, 30, 30 and 43m<br>• 1 lost to follow-up	1 free from progression and alive after 18m
Düzköylü Y., 2018	10	15m	• 6 alive after 3, 10, 20, 26, 31, and 51m<br>• 3 died after 3, 10 and 22m	1 died after 5m
La Rosa S., 2018	14	6.5 yrs	• 12 free from recurrence/progression and alive after 1, 1, 2, 2, 2, 4, 9, 11, 12, 12, 24 and 27 yrs<br>• 2 lost to follow-up	
Dulskas, A. 2019	9	17m	• 3 alive after 20, 28 and 56m	• 2 alive after 11 and 18m<br>• 4 died after 2, 11, 14 and 17m

yrs = years; m = months; N= number.

Table S2. Comparison of survival outcomes of mixed neuroendocrine non-neuroendocrine neoplasms with survival outcomes of other neoplasms from the same sites of origin.

Reference	Site	MiNEN vs NEC	MiNEN vs NET	MiNEN vs GCC
Shen C., 2016	Stomach	In patients with radical resection, no difference in OS (p=0.102)	In patients with R0 resection, WD-NET have better OS (p<0.001)	
Lim S.M., 2016	Stomach	No difference in OS (p=0.232)		
Park J.Y., 2014	Stomach	No difference in RFS or in OS (p = not provided)		
van der Veen A., 2018	Stomach and oesophagus/OGJ	No difference in OS (p=0.403)		
Yang M., 2015	Pancreas	No difference in OS (p=0.071)	WD-NET have better OS than NEC/MiNEN (p<0.001)	
Basturk O., 2014	Pancreas	MiNEN have better OS than NEC (p=0.0035)		
Zheng Z., 2019	Biliary tract	No difference in RFS (p=0715) or OS (p=0.670)		
Brathwaite S., 2016	Appendix		WD-NET have better OS than NEC/MiNEN (p<0.001)	GCC have better OS than MiNEN (p<0.001)
Brathwaite S., 2016	GEP		WD-NET have better OS than NEC/MiNEN (p=0.007)	Trend (although not statistically significant, p=0.52) towards a better OS for GCC (13.2 years) compared to MiNEN (4.1 years)
Milione M., 2018	GEP	No difference in OS (p=0.08)		

MiNEN = mixed neuroendocrine non-neuroendocrine neoplasm; NEC = pure neuroendocrine carcinoma; NET = well differentiated neuroendocrine tumour; GCC = goblet cell carcinoid; WD = well differentiated; OS = overall survival; RFS = recurrence free survival; OGJ = oesophagogastric junction; GEP = gastroenteropancreatic tract.
